# A Radiomics Nomogram for Preoperative Prediction of Early Recurrence of Small Hepatocellular Carcinoma After Surgical Resection or Radiofrequency Ablation

**DOI:** 10.3389/fonc.2021.657039

**Published:** 2021-04-29

**Authors:** Liting Wen, Shuping Weng, Chuan Yan, Rongping Ye, Yuemin Zhu, Lili Zhou, Lanmei Gao, Yueming Li

**Affiliations:** ^1^ Department of Radiology, The First Affiliated Hospital of Fujian Medical University, Fuzhou, China; ^2^ Department of Radiology, Fujian Maternity and Child Health Hospital, Affiliated Hospital of Fujian Medical University, Fuzhou, China; ^3^ Key Laboratory of Radiation Biology, Fujian Medical University, Fujian Province University, Fuzhou, China

**Keywords:** hepatocellular carcinoma, radiomics, nomogram, recurrence, magnetic resonance imaging

## Abstract

**Background:**

Patients with small hepatocellular carcinoma (HCC) (3 cm) still have a poor prognosis. The purpose of this study was to develop a radiomics nomogram to preoperatively predict early recurrence (ER) (2 years) of small HCC.

**Methods:**

The study population included 111 patients with small HCC who underwent surgical resection (SR) or radiofrequency ablation (RFA) between September 2015 and September 2018 and were followed for at least 2 years. Radiomic features were extracted from the entire tumor by using the MaZda software. The least absolute shrinkage and selection operator (LASS0) method was applied for feature selection, and radiomics signature construction. A rad-score was then calculated. Multivariable logistic regression analysis was used to establish a prediction model including independent clinical risk factors, radiologic features and rad-score, which was ultimately presented as a radiomics nomogram. The predictive ability of the nomogram was evaluated using the area under the receiver operating characteristic (ROC) curve and internal validation was performed *via* bootstrap resampling and 5-fold cross-validation method.

**Results:**

A total of 53 (53/111, 47.7%) patients had confirmed ER according to the final clinical outcomes. In univariate logistic regression analysis, cirrhosis and hepatitis B infection (*P*=0.015 and 0.083, respectively), hepatobiliary phase hypointensity (*P*=0.089), Child-Pugh score (*P*=0.083), the preoperative platelet count (*P*=0.003), and rad-score (*P*<0.001) were correlated with ER. However, after multivariate logistic regression analysis, only the preoperative platelet count and rad-score were included as predictors in the final model. The area under ROC curve (AUC) of the radiomics nomogram to predict ER of small HCC was 0.981 (95% CI: 0.957, 1.00), while the AUC verified by bootstrap is 0.980 (95% CI: 0.962, 1.00), indicating the goodness-of-fit of the final model.

**Conclusions:**

The radiomics nomogram containing the clinical risk factors and rad-score can be used as a quantitative tool to preoperatively predict individual probability of ER of small HCC.

## Introduction

Liver cancer was the sixth most frequently diagnosed cancer and the fourth most common cause of cancer death globally in 2018, while hepatocellular carcinoma (HCC) accounted for 75-85% of cases (1). With the development of diagnostic equipment and advances in diagnostic techniques, more HCCs can be detected at an early stage (single tumor of 3cm or less) (2, 3). According to the American Association for the Study of Liver Diseases (AASLD) and European Association for the Study of Liver (EASL) management guidelines, the recommended treatment options for HCC include surgical resection (SR), radiofrequency ablation (RFA), and liver transplantation (4). Currently, SR and RFA are often used as curative treatment of small HCC (diameter of single cancer nodules 3cm, or the sum of diameter of two cancer nodules 3cm) (5). Unfortunately, even small HCC patients may have a poor prognosis due to the high incidence of tumor recurrence and metastasis (6,8). While according to the recent clinical practice guideline of HCC, recurrence of HCC is classified as either early recurrence (ER) (less than 2 years) or late recurrence (more than 2 years) (7, 8). In addition, HCC with ER generally has a poorer prognosis (9). ER is often considered to be the result of occult metastasis of the primary tumor (9, 10).

Numerous studies have shown that ER is associated with tumor aggressiveness, including tumor size, poor-cell differentiation, microscopic and macroscopic vascular invasion, and some blood indicators (11,13). Currently, there are many investigators attempting to predict ER by using conventional magnetic resonance imaging (MRI), apparent diffusion coefficient (ADC) maps, diffusion kurtosis imaging (DKI) (14,16). However, some of these sequences often require additional acquisitions and are susceptible to subjective factors. Therefore, ER remains one of the major obstacles to improving patients outcomes due to the lack of an objective and reliable preoperative prediction tool.

Recently, radiomics has been widely used to capture tumor heterogeneity by extracting and evaluating quantitative features from digital medical images for the assessment of tumor aggressiveness and prognosis (17, 18). In the field of HCC, radiomics has been used as a noninvasive tool to predict ER by comparing differences in texture parameters, or building a comprehensive classification model (19, 20). However, these studies only looked at ER after SR, omitting patients who underwent RFA. RFA is often used as first-line therapy with the advantages of minimal invasive, few complications and short hospital staying for small HCC. The long-term overall survival and tumor-free survival are thought to be not significantly different between SR and RFA (21). Furthermore, to the best of our knowledge, there have been few previous studies on the relationship between MRI-based radiomics signatures and the ER of small HCC after SR or RFA.

Therefore, this study was aimed to establish a radiomics nomogram to preoperatively predict ER of small HCC and to further provide the clinician with a quantitative tool for predicting individual probability of ER.

## Materials and Methods

### Patients

**Figure 1:**
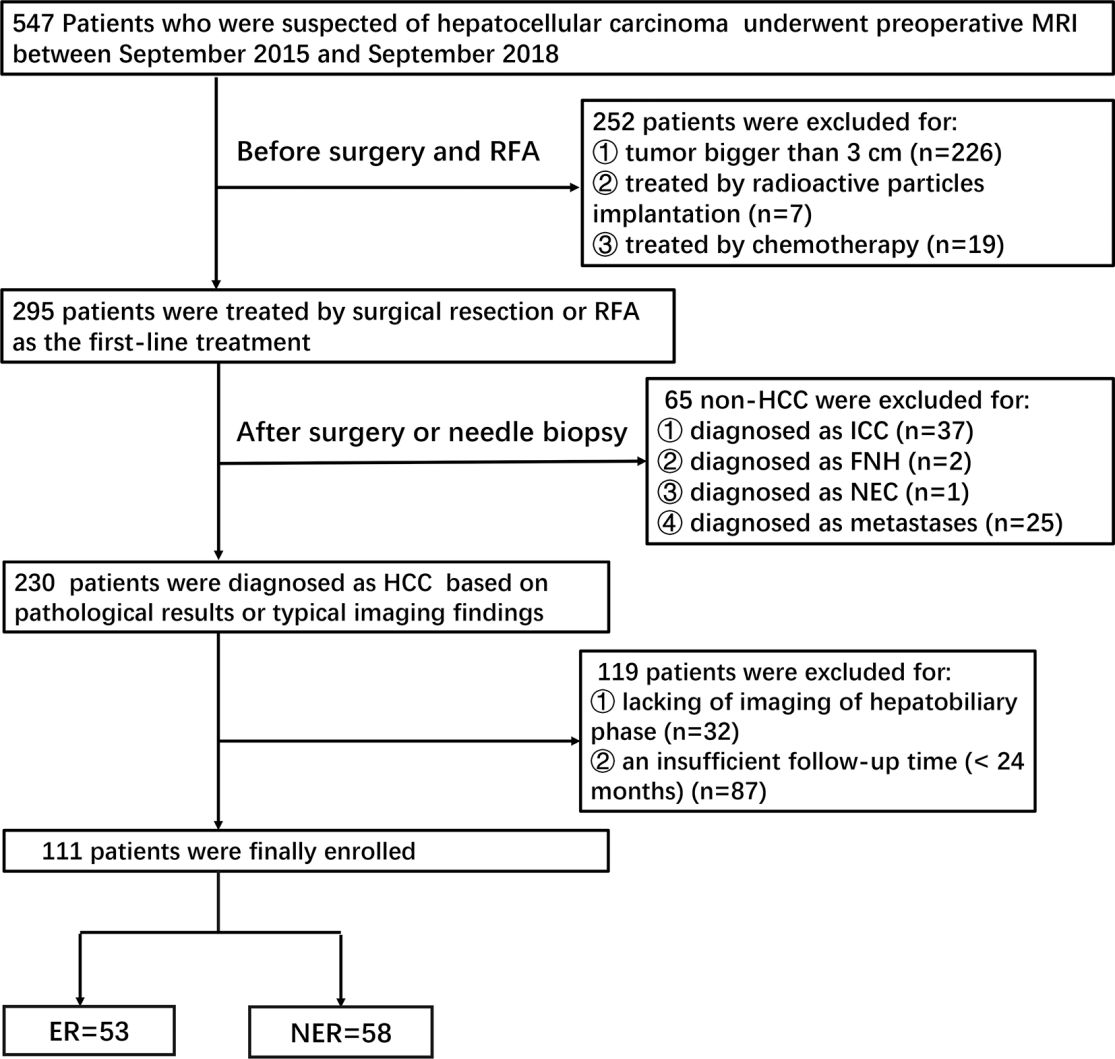
Flowchart showing the inclusion and exclusion of patients.

This retrospective study was approved by the institutional review board of our hospital, and the requirement of informed consent was approved for waiver. Patients were identified by searching through the picture archiving and communication system (PACS) database between September 2015 and September 2018. Preoperative gadobenate dimeglumine enhanced MRI were performed on 547 patients who were suspected of HCC. Two hundred and forty-two patients were initially excluded before SR or RFA due to tumor size (> 3cm) (**Figure 1**).

Patients were subsequently included according to the following criteria: 1) Patients with HCC confirmed by pathology or typical imaging findings (significant enhancement on arterial phase and wash-out in the portal venous or delayed-phase in multiphasic MRI); 2) Patients who underwent MRI examination within one month prior to SR or RFA; 3)Patients with complete clinical and laboratory data.

Exclusion criteria were as following: 1) Histopathologically diagnosed as other tumors rather than HCC (n=65); 2) Patients who underwent other treatments prior to SR or RFA (n=26); 3) Poor MR image quality, or lack of hepatobiliary imaging (n=32); 4) An insufficient follow-up time (<24 months) (n=87).

Patients were followed up regularly every 2-3 months for 2 years after SR or RFA and were monitored for recurrence by standard parameters including alpha-fetoprotein (AFP) level, liver function, CT and especially MRI. ER was defined as a new lesion with typical imaging characteristics in the remnant liver or other organs within 2 years after SR or RFA. Patients were followed until either ER or the end date of this study (September 30, 2020).

### Clinical Characteristics

Clinical characteristics before SR or RFA were retrospectively obtained from an electronic medical record system. Demographic characteristics, history of hepatitis and cirrhosis, Child-Pugh score, AFP, alanine aminotransferase (ALT), aspartate aminotransferase (AST), and preoperative platelet count (PLT) were analyzed.

### MRI Data Acquisition

One month prior to SR or RFA, MRI was performed in all patients by using a 3.0 T MRI scanner (MAGNETOM Verio; Healthineers, Erlangen, Germany) with a dedicated phased-array body coil. The standard abdominal MRI protocol consisted of the following sequence: 1) axial T2-weighted fat-suppressed turbo-spin-echo (TSE): repetition time (TR)/echo time (TE), 4700/79 msec, slice thickness, 5mm, slice gap, 1mm, FOV, 21 mm38 mm; 2) DWI (b=50, 800 sec/mm^2^) performed with a free-breathing single-shot echo-planar technique, TR/TE, 9965/73 msec, slice thickness, 5mm, slice gap, 1mm, FOV, 21 mm38 mm. Corresponding ADC maps were automatically calculated by the MRI system; 3) multiphase contrast enhanced MRI, a 3D gradient echo sequence with volumetric interpolated breath-hold examination (VIBE), was performed before and after injection of Gadobenate Dimeglumine (MultiHance; Bracco, Shanghai, China) 0.2 ml/kg at a rate of 2 ml/sec followed by a 20ml saline flush with the following parameters: TR/TE, 3.9/1.4 msec, slice thickness, 3mm, slice gap, 1mm, FOV, 25 mm80 mm. Hepatic arterial phase (AP), portal venous phase (PVP), equilibrium phase images and hepatobiliary phase (HBP) were obtained at 2030 sec, 7080 sec, 180 sec and 90min after contrast medium injection, respectively.

### Imaging Feature Evaluation

All MR images were independently reviewed by two experienced radiologists to assess the imaging features of the HCC in a single blind manner (unknown treatment for the patients and whether there were ER after SR or RFA). The two radiologists met to make final decisions by consensus when discordant cases occurred. The imaging features were selected according to the Liver Imaging Reporting and Data System (LI-RADS 2018) diagnostic algorithm including major features (nonrim arterial phase hyperenhancement (APHE), nonperipheral washout appearance (washout), enhancing capsule appearance (capsule), size and ancillary features (mild-moderate T2 hyperintensity, restricted diffusion, hepatobiliary phase hypointensity, etc.) (22). The maximum diameter of the tumor was measured at the level of the maximum cross-sectional area (if the lesion was clearly visible in other phases, do not measure in the arterial phase and DWI were avoided due to lesion size overestimation.

### Feature Selection and Radiomics Signature Building

MaZda software (version 4.6, available at http://www.eletel.p.lodz.pl/mazda/) was used for texture analysis (TA) [24,25]. All MRI were transformed into Digital Imaging and Communications in Medicine (DICOM) format for compatibility.

All tumors were manually delineated by observer 1 (a radiologist with 7 years of experience in abdominal imaging), and the region of interest (ROI) was plotted on each cross section of the entire lesion. Texture features of the tumor were extracted from the T1 weighted, T2 weighted, AP, PVP and HBP image. HBP or T2 weighted imaging (in case of artifact) were used as the reference to delineate the tumor and were first segmented. Subsequently, the ROI was overlaid onto other phase images as required. If the tumor location had changed due to respiratory movement, fine adjustments were made to the ROI. The result of 101 features (derived from histogram, the absolute gradient, Gray Level Run-Length Matrix, Gray Level Co-occurrence Matrix, autoregressive model and wavelet transform) were generated from each three-dimensional segmentation, giving a total of 505 features for every lesion.

Twenty-six patients were randomly selected (12 ER and 14 NER) to explore the stability of each feature; observer 1 repeated tumor segmentation and observer 2 (with 9 years of experience in liver imaging) independently performed the segmentation in five image sequences (T2WI, T1WI, AP, PVP and HBP sequences). Intraclass and intergroup correlation coefficient (ICC) were used to evaluate the intra- and inter-observer repeatability of radiomics features, respectively. There is a good agreement of the feature extraction when the ICC is greater than 0.8.

To avoidoverfitting and the curse of dimensionality, all radiomics features were loaded into the MAZDA feature selection package by image sequence (T2WI, T1WI and AP, PVP and HBP sequences) to select the most discriminative radiomics features between the ER group and the NER group. Feature selection algorithms included Mutual information [MI], fisher coefficient [Fisher], and classification error probability combined with average correlation coefficients [POE + ACC]. The three feature selection methods were supervised methods. Based on the MaZdas automatic techniques, they were combined for the identification of 150 texture features in total, with the highest discriminative power for classification.

The least absolute shrinkage and selection operator (LASSO) method, which is suitable for the regression of high-dimensional data, was used to select the most useful predictive features among the final 150 texture features. LASSOis a regularization algorithm which can be used to eliminate irrelevant noises and do feature selection. It heavily relies on parameter , which is the controlling factor in shrinkage. The optimal value of is found by 10-fold cross-validationand 100 replicatesto find the minimum mean squared error (minMSE) or minMSE + 1 standard error of minMSE backwards along the path (minMSE + 1SE), i.e., the largest -value such that the error is within 1SE of the minimum.The larger becomes, then the more coefficients are forced to be zero. Coefficients of some irrelevant variablesare imposedto shrink towards zero,and every variable of which coefficient is non-zero is selected as the most significant predictor to be used in the model.

### Development of an Individualized Prediction Model

A combined model was created after analyzing potential factors in multivariate logistic regression analysis which included independent clinical risk factors, radiologic features and radiomics score. The nomogram based on the combined model was established to provide the clinician with a quantitative tool to predict individual probability of ER. To quantify the prognostic performance, area under the curve (AUC) for receiver operating characteristic (ROC) curve for the prediction model was then calculated with 95% confidence intervals (CIs). The optimal cutoff values from the maximum Youdens index, as well as the corresponding sensitivity and specificity for discriminating ER and NER, were obtained from ROC curve analysis. The model was internally validated using 1000 bootstrap samples to avoid overfitting. Another validation of 5-fold cross-validation was also applied. The calibration curve provided a comparison between the expected and observed conversion probabilities.

### Statistical Analysis

Continuous variables were expressed as the mean standard deviation. The two-sample t-test or the MannWhitney U test for continuous variables, whereas the chi-square test or Fishers exact test was used as appropriate to compare the differences in categorical variables. Univariate and multivariate logistic regression analyses were performed to screen the independent risk factors of ER. Factors with a *p* value of 0.10 or less at univariate analyses were entered into the multivariate model. Odds ratios and 95% CIs were calculated. Model performance was assessed by model calibration. A *p*-value less than 0.05 (typically 0.05) is statistically significant. All statistical analyses were performed by using SPSS software (version 25.0; SPSS, Chicago, Ill) and R statistical software (version 3.6.3, https://www.r-project.org/).

## Results

### Patient Characteristics

A total of 111 patients including 93 (83.8%) male and 18 (16.2%) female were finally enrolled. These small HCC patients were treated with SR (n=45) or RFA (n=66) as initial therapy. A total of 53 (53/111, 47.7%) patients had confirmed tumor recurrence according to the two-year follow-up outcomes. Subsequently, patients were divided into the ER group (n=53) and the non-early recurrence (NER) group (n=58). Comparisons of baseline characteristics between small HCCs with or without ER are summarized in **Table 1**. There was no difference in the rate of ER between SR and RFA (*P*=0.336).

**Table 1:** Baseline characteristics of ER and NER groups in the study population.

Parameter	ER (n=53)	NER (n=58)	*p*
Age(y)*	53.18 12.60	55.62 11.61	0.294
Sex			0.759
male	45 (84.9)	48 (82.8)	
female	8 (15.1)	10 (17.2)	
HBsAg			0.079
Positive	49 (92.4)	47 (81.0)	
Negative	4 (17.6)	11 (19.0)	
Liver cirrhosis			0.182
present	44 (83.0)	42 (72.4)	
absent	9 (17.0)	16 (27.6)	
AFP>20ng/ml			0.078
present	28 (52.8)	21 (36.2)	
absent	25 (47.2)	37 (63.8)	
ALT(U/L) *	57.56 118.11	85.84 201.26	0.303
AST(U/L) *	57.32 134.95	81.78 216.35	0.395
ALT/AST *	1.07 0.423	1.11 0.346	0.573
Preoperative PLT	117.85 77.61	157.78 77.61	**0.004**
Treatment			0.336
Surgical Resection	19 (35.8)	26 (44.8)	
Radiofrequency Ablation	34 (64.2)	32 (55.2)	

Unless otherwise indicated, data are numbers of patients, with percentage in parentheses.

*Data are means standard deviation. P values that are statistically significant are in bold.

ER, Early recurrence; NER, None early recurrence; ALT, Alanine aminotransferase level; AST, Aspartate aminotransaminase level; AFP, alpha-fetoprotein; PLT, Platelet count.

**Figure 2:**
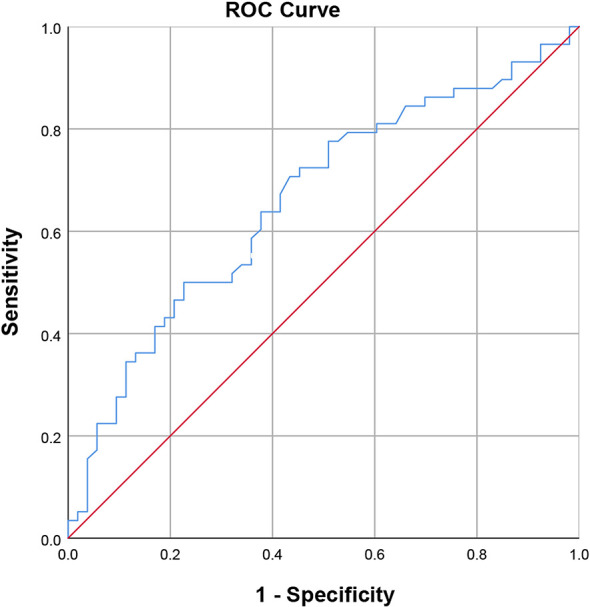
Receiver operating characteristic curve of the preoperative platelet count (area under the curve = 0.661, standard error = 0.052, *p* = 0.004, 95% confidence interval = 0.559-0.762).

There were significant differences in preoperative platelet count between the ER group and NER group (*P*=0.004). However, the other baseline characteristics did not differ between the two groups significantly. The optimal cut-off value was 157.510^3^/ml, corresponding to the maximum sensitivity and specificity of the ROC curve, where the AUC of the platelet count was 0.661 (**Figure 2**). Patients with low preoperative platelet counts (<157.510^3^/ml) were significantly more likely to recur than those with high preoperative platelet counts.

### MRI Feature Evaluation

The conventional MR imaging features between the ER and NER groups were described in **Table 2**. We found that the enhancing capsule was statistically different between ER group and NER group (*P*=0.019). However, no significant differences were detected between the two groups across other imaging features, including tumor size, nonrim APHE, nonperipheral washout appearance, restricted diffusion, mild-moderate T2 hyperintensity, hepatobiliary phase hypointensity.

**Table 2:** Analysis of radiologic features between ER and NER of small HCCs.

Parameter	ER (n=53)	NER (n=58)	*p*
Tumor size(cm)*	1.730.61	1.760.64	0.795
Nonrim APHE			0.185
present	44 (83.0)	53 (91.4)	
absent	9 (17.0)	5 (8.6)	
Washout			0.112
present	36 (67.9)	47 (81.0)	
absent	17 (32.1)	11 (19.0)	
Enhancing capsule			**0.019**
present	23 (43.4)	38 (65.5)	
absent	30 (56.6)	20 (34.5)	
Restricted diffusion			0.096
present	39 (73.6)	50 (86.2)	
absent	14 (26.4)	8 (13.8)	
Mild-moderate T2 hyperintensity			0.069
present	42 (79.2)	53 (91.4)	
absent	11 (20.8)	5 (8.6)	
Hepatobiliary phase hypointensity			0.289
present	45 (84.9)	53 (91.4)	
absent	8 (15.1)	5 (8.6)	

Unless otherwise indicated, data are numbers of patients, with percentage in parentheses.

*Data are meansstandard deviation. P values that are statistically significant are in bold.

HCC, Hepatocellular carcinoma; ER, Early recurrence; NER, None early recurrence; APHE, Arterial phase hyperenhancement.

### Feature Selection and Radiomics Signature Building

The interobserver ICC was >0.8, 0.5-0.79, <0.5 for 98%, 1% and 1% of the radiomic features extracted from five images sequences, respectively. The intraobserver ICC was >0.8, 0.5-0.79, <0.5 for 94%, 4% and 1% of the radiomic features extracted from six images sequences, respectively.

Nine potential features were selected by LASSO algorithm. These features were presented in the rad-score calculation formula. Features derived from non-enhanced T1 weighted images were reduced to 0, meaning a pretty poor robustness. The selected features in T2 weighted images included S(0, 0,1)Correlat. In AP images, the selected features included S(0,0,1)Correlat, Perc.01%3D, S(1,1,0)InvDfMom, GrNonZeros, S(0,1,0)InvDfMom, S(1,0,0)Correlat. And S(1,1,0)SumAverg in PVP images, Skewness3D in HBP images were also included. The rad-score for individual patients was calculated using the following formula: Rad-score = 5.670 + 0.487 S(0, 0,1)Correlat (T2) - 5.272 S(0,0,1)Correlat (AP) - 0.001 Perc.01%3D(AP) - 4.071 S(1,1,0)InvDfMom(AP) - 1.010 GrNonZeros(AP) - 4.242 S(1,0,0)Correlat(AP) - 0.901 S(1,0,0)Correlat(AP) + 0.001 S(1,1,0)SumAverg(PVP) + 5 10^-5^ Skewness3D(HBP). There were significant differences in rad-score between ER and NER patients (*P*=0.001), patients with ER generally presented higher rad-scores.

The area under the ROC curve of the rad-score was 0.979 and the optimal cutoff value was -6.15.

### Development of Individualized Predictive Models

**Figure 3:**
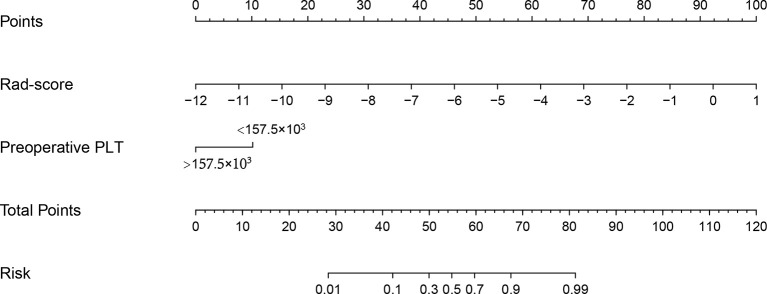
Radiomics nomogram developed with the rad-score and the preoperative platelet count. The nomogram is valued to obtain the probability of ER by adding up the points identified on the points scale for each variable.

**Table 3** summarized the risk factors found to be associated with ER in small HCC after univariate analysis, including the presence of liver cirrhosis (*P*=0.015), hepatitis B virus infection (*P*=0.083), presence of hepatobiliary phase hypointensity (*P*=0.089), the Child-Pugh score (*P*=0.083), preoperative platelet count (*P*=0.003), and the rad-score (*P*=0.001). No multicollinearity was found among all independent variables in the multivariate analysis. Eventually, multivariate logistic regression analysis further identified the rad-score and preoperative platelet count as the final independent predictors of ER of small HCC. The model that incorporated the aforementioned independent predictors was developed and further presented in the form of a nomogram (**Figure 3**). The nomogram was used to provide clinicians with a quantitative tool for predicting the individual probability of ER.

**Table 3:** Univariate and multivariate logistic regression analyses of the risk factors for ER of HCC.

Variable	Univariate Analysis		Multivariate Analysis	
	Odds Ratio	*P* Value	Odds Ratio	*P* Value
Age (y)	1.02 (0.99-1.05)	0.292		
AFP>20ng/ml	1.58 (0.74-3.36)	0.234		
liver cirrhosis HbsAg Child-Pugh class	3.53 (1.28-9.73) 2.87 (0.85-9.64) 0.43 (0.17-1.12)	0.015 0.089 0.083		
ALT (U/L)	1.00 (1.00-1.00)	0.398		
AST (U/L)	1.00 (1.00-1.00)	0.497		
ALT/AST	0.75 (0.28-2.01)	0.570		
Preoperative PLT Tumor >2.0cm	0.29 (0.13-0.67) 0.70 (0.31-1.55)	0.003 0.375	0.17 (0.03-0.99)	0.049*
Nonrim APHE	0.46 (0.14-1.48)	0.193		
Washout	0.50 (0.21-1.19)	0.115		
Enhancing capsule	1.46 (0.68-3.14)	0.336		
Hepatobiliary phase hypointensity	0.36 (0.12-1.12)	0.077		
Radiomics signature	4.09 (1.74-9.6)	0.001	3.80 (1.70-8.5244)	0.001*

HCC, Hepatocellular carcinoma; ER, Early recurrence; NER, None early recurrence; ALT, Alanine aminotransferase level; AST, Aspartate aminotransaminase level; AFP, alpha-fetoprotein; PLT, Platelet count; APHE, Arterial phase hyperenhancement.

*p < 0.05.

**Figure 4:**
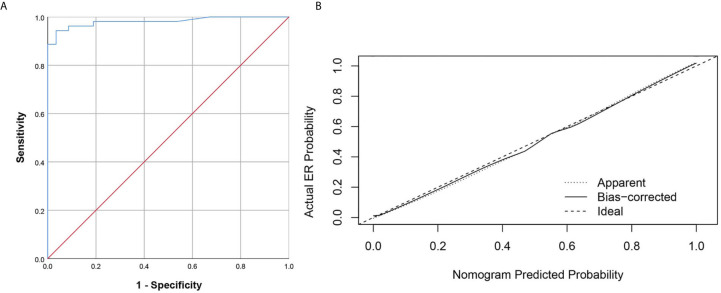
The ROC curve and calibration curves for the radiomics nomogram. **(A)** Graph shows utility of ROC curve of the radiomics nomogram to discriminate ER and NER of small HCC. **(B)** Calibration curves for the radiomics nomogram. Calibration curves indicate the goodness-of-fit of the nomogram. The closer the full line approaches the ideal prediction line, the better the predictive efficacy of the nomogram.

**Figure 5:**
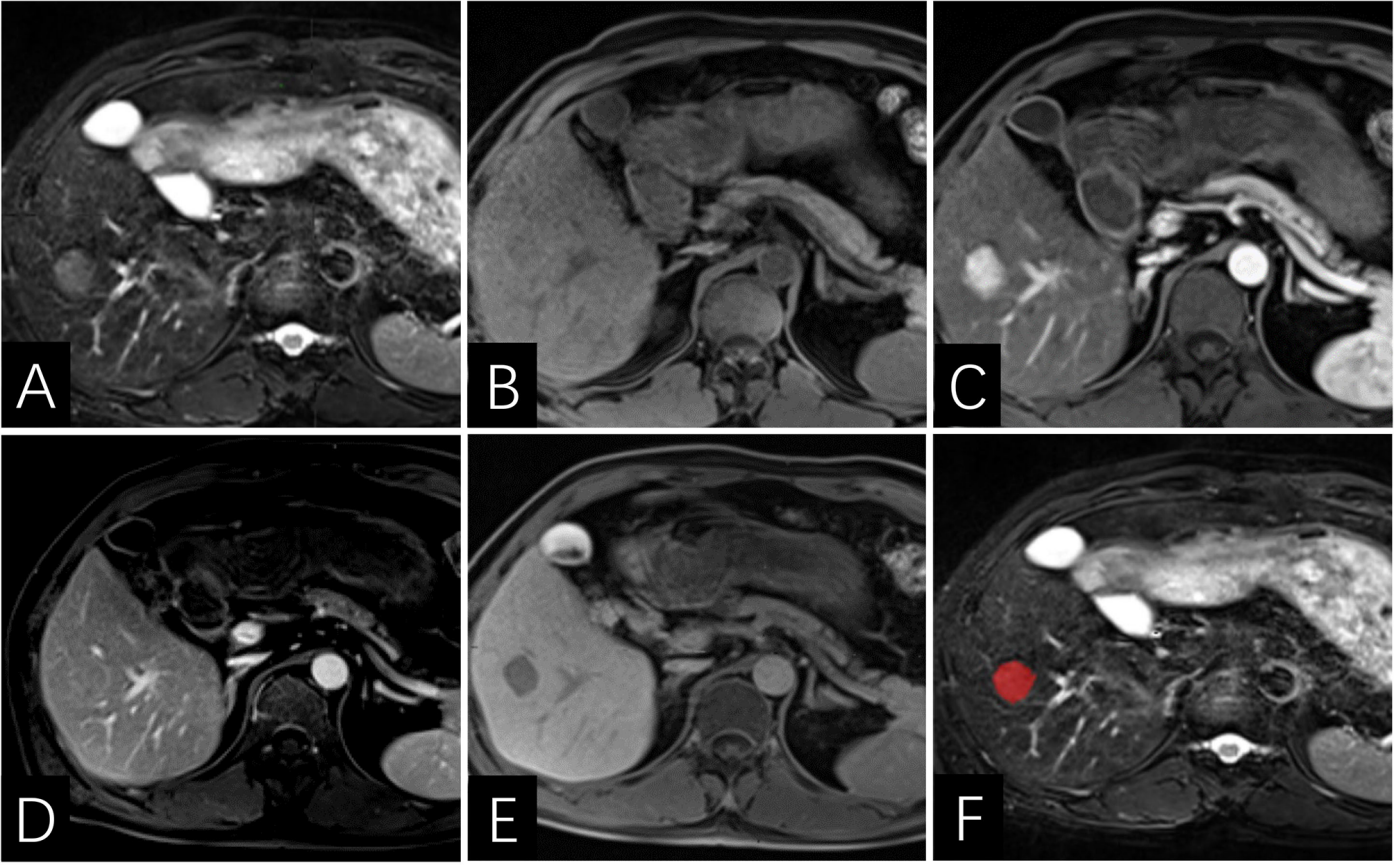
Images of a 46-year-old man with HCC without early recurrence. **(AE)** the tumor demonstrates lack of enhancing capsule with hepatobiliary phase hypointensity. **(F)** the lesion was first ROI segmented in red and the ROI was plotted on each cross section of the entire lesion to get the texture information. The Rad-score of this patient was -6.7, and his PLT was 20910^3^/ml. According to the Nomogram, his total point was about 44, indicating the risk of ER was more than 0.1 but less than 0.3.

**Figure 6:**
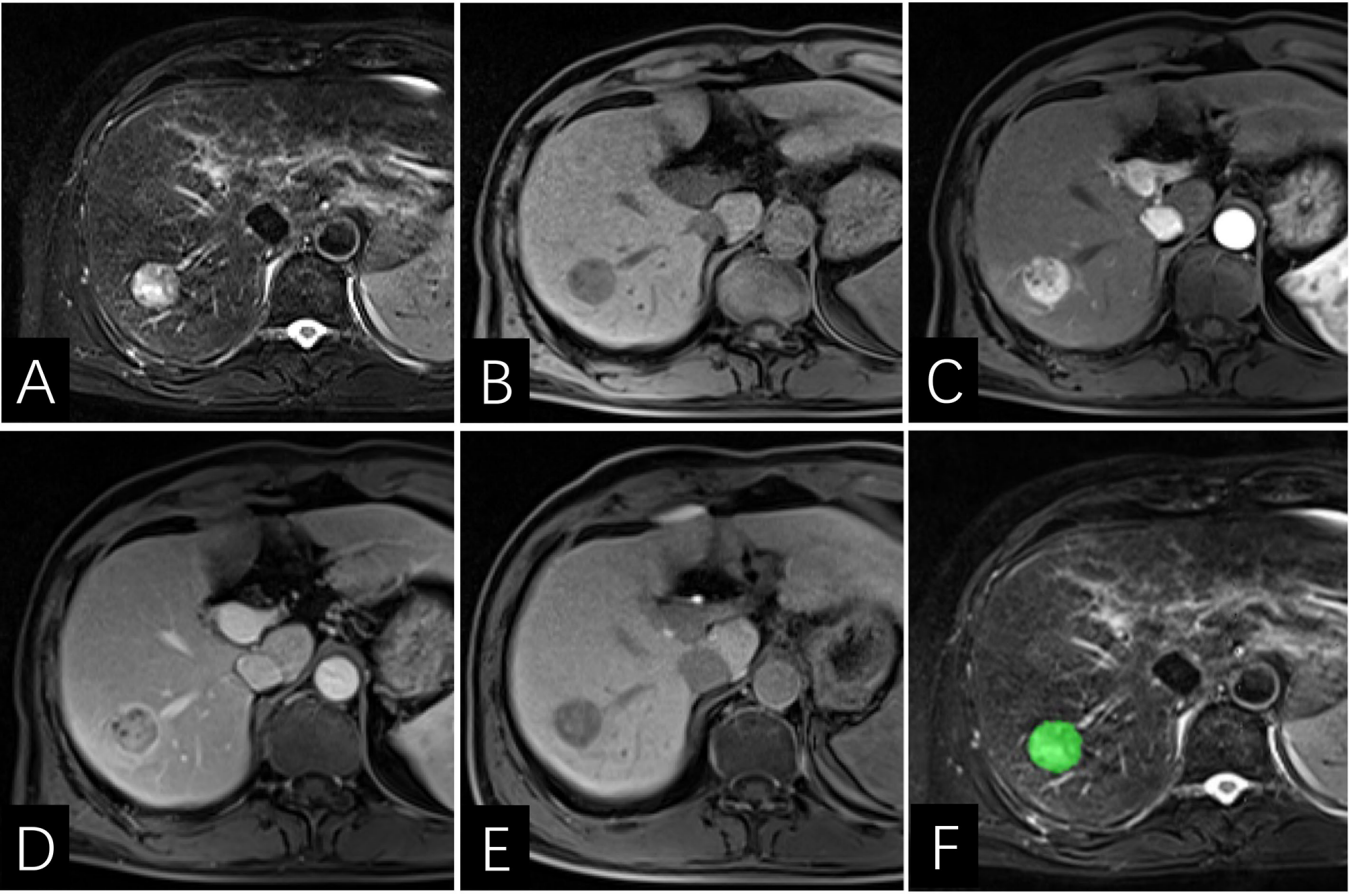
Images of a 64-year-old man with HCC with early recurrence. **(AE)** the tumor displays enhancing capsule and hepatobiliary phase hypointensity. **(F)** the lesion was first ROI segmented in green and the ROI was plotted on each cross section of the entire lesion to get the texture information. The Rad-score of this patient was -5.2, and his PLT is 14410^3^/ml. Based on the Nomogram, the patients total score was about 67, indicating that the risk of ER was between 0.8-0.9.

The prediction ability of the final model was assessed using the AUC (estimated to be 0.981, 95% CI:0.957, 1.000, standard error 0.012) (**Figure 4A**) as well as the bias-corrected AUC, which was estimated using bootstrap with 1000 iterations and noted to be 0.980 (95% CI: 0.962, 1.000). The result of internal 5-fold cross-validation (AUC: 0.968, 95% CI: 0.916, 0.992, standard error 0.019) also showed favorable predictive efficacy. **Figure 4B** showed the calibration curve of the nomogram. The ideal curve fitted well with the calibration prediction curve, indicating the goodness-of-fit of the nomogram. Two case were provided to show nomograms ability to predict ER (**Figures 5** and **6**).

The Nomo-score was calculated using the following formula: Nomo-score = 8.277 + radscore 1.336 PLT(1, <157.510^3^; 0, >157.510^3^) 1.767

## Discussion

Treatments for small HCC remain to be plagued by high recurrence rate, as evidenced by the observance that nearly half of the patients in our study developed tumor recurrence (53 in 111 patients). Thus, the development of any powerful tool to predict the ER of small HCC is promising. This study established a radiomics nomogram based on preoperative MRI, including the preoperative platelet count and radiomics signature (rad-score). The area under the ROC curve for radiomics nomogram prediction of ER of small HCC was 0.981 (95% CI: 0.957, 1.00). The radiomics nomogram, developed herein, had high prediction power, allowing clinicians to skip complex calculations and simply use the preoperative factors. Additionally, this nomogram can provide a reference for closer follow-up or more aggressive treatment for patients who are predicted to be ER positive.

So far, there have been many studies on the ER of HCC (23,25). In contrast to previous studies, the current study constructed a radiomics nomogram model to assess the individualized prediction of recurrence risk. The radiomics model presented here was based on preoperative MRI examination, which could be more objective and highly reproducible. Huang Z and his colleagues (26) found that nomogram models can be useful in determining the risk of recurrence-free survival with a C-index of 0.733 (95%CI: 0.672, 0.774). Zhang X etal. (27) constructed a CT radiomics-based models to predict microvascular infiltration (MVI) status and MVI risk of HCC. They concluded that the radiomics-based models presented as a reliable preoperative evaluation tool, with an AUC of 0.746 for CT radiography-based models. In this study, the radiomics nomogram also showed a good prediction of efficiency (AUC=0.981).

Compared with the basic MR imaging features, radiomics can objectively and quantitatively capture more information about intra-tumor heterogeneity based on pixel gray values (20, 28, 29), however, there is still controversy over the best method to obtain ROI. Ng F etal. (30) were in favor of abstracting the entropy and uniformity of whole tumor rather than using the largest cross-sectional area for the analysis of survival, because the analysis of the whole tumor was more representative of tumor heterogeneity. Gourtsoyianni etal. (31) also found that it was better to use global textural parameters in rectal cancer in terms of MRI repeatability. On the basis of previous studies, we chose to incorporate features of the whole tumor. Linear calculation was used to formulate a rad-score for each patient: the rad-scores demonstrated strong statistical significance in univariate and multivariate logistic analyses between ER and NER groups (*P*=0.001, both).

Note that the preoperative platelet count was the other independent risk factors for ER (P=0.049). However, there is little data to assess the prognostic value of preoperative PLT in patients with HCC. As we know, HCC usually occurs in patients with cirrhosis, and decreased platelet count is common due to portal hypertension and splenic isolation (4, 32). Pang Q etal. (33) reported that a low preoperative PLT level results in an unfavorable outcome in HCC. Ahmed Shehta etal. (34) also demonstrated that thrombocytopenia was a significant predictor of HCC recurrence after liver resection. In contrast, other studies concluded that thrombocytosis was a predictor of HCC recurrence reasoned due to the fact that blood platelets produce inflammatory mediators which play active roles in angiogenesis and tumor metastasis (35, 36). Furthermore, antiplatelet therapy can reduce the ER of HCC (37), and improve patients survival (38). In our study, the preoperative platelet count was strongly associated with recurrence of small HCC. Recurrence rate increased at lower platelet counts, and decreased at higher platelet counts. Further studies should be performed to more precisely characterize the mechanism between the platelet count and the recurrence of HCC.

Other factors such as age, AFP, PIVKA-II, tumor grade, tumor size, peri-tumor parenchymal enhancement in the arterial phase, unsmooth tumor margins, peri-tumor hypointensity in the hepatobiliary phase, and ADC values have been reported to be significantly associated with early tumor recurrence (39, 40). However, some of these clinic-radiologic factors were not found to be statistically significant in our study. Such inconsistency in existing studies may be related to the fact that all the cases we studied were small HCC. Some studies (41) have shown that small hepatocellular carcinoma does not exhibit typical imaging findings. Nevertheless, there are several limitations to our study. First, this was a retrospective single-center study design, which, inevitably, may have introduced selection bias. Second, 66 nodules were treated by RFA without obtaining histopathologic evidence. Despite this, we followed the practice guidelines of the American Association for the Study of Liver Diseases (4), which state that patients with typical imaging features of HCC (>1cm) at four-phase dynamic CT or MR imaging do not require further evaluation to confirm the presence of HCC, and further initiation of appropriate therapy is recommended. Third, we did not use internal validity or external validity to check the generalizability, which may lead to data overfit. In future studies, a larger cohort population should be included to validate the results. Last but not least, this study only focused on intratumoral radiomics and some clinical parameters, ignoring the peritumoral region. Peritumoral ROI should be performed in the future to extract more radiomics features.

In conclusion, a radiomics nomogram was constructed to execute a preoperative prediction of the ER after SR or RFA for small HCC. This radiomics nomogram could be helpful for preoperative clinical decision-making of small HCC.

## Data Availability Statement

The raw data supporting the conclusions of this article will be made available by the authors, without undue reservation.

## Ethics Statement

This single-center retrospective cohort study was approved by the Institutional Review Board of the First Affiliated Hospital of Fujian Medical University who determined the requirement for informed consent could be waived.

## Author Contributions

Conception and design: YL, LW, and SW. Development of methodology: YL and SW. Acquisition of data: LW, CY, RY, YZ, LZ, and LG. Analysis and interpretation of data: LW, CY, JC, and RY. Editing and review of the manuscript: all authors. Study supervision: YL and SW. All authors contributed to the article and approved the submitted version.

## Funding

This study has received funding by Joint Funds for the Innovation of Science and Technology, Fujian province (CN) (Award Number: 2019Y9125).

## Conflict of Interest

The authors declare that the research was conducted in the absence of any commercial or financial relationships that could be construed as a potential conflict of interest.

## Abbreviations

HCC, Hepatocellular carcinoma; SR, Surgical resection; RFA, Radiofrequency ablation; ER, Early recurrence; Rad-score, Radiomics score; LASSO, Least absolute shrinkage and selection operator; AUC, Area under the curve; ROC, Receiver operating characteristic; ROI, Region of interest; Nomo-score, Nomogram score; TIWI, T1-weighted imaging; T2WI, T2-weighted imaging; DWI, Diffusion weighted imaging; AP, Arterial phase; PVP, Portal venous phase; HBP, Hepatobiliary phase; LI-RADS, Liver Imaging Reporting and Data System; APHE, Arterial phase hyperenhancement.
